# Marine foods sourced from farther as their use of global ocean primary production increases

**DOI:** 10.1038/ncomms8365

**Published:** 2015-06-16

**Authors:** Reg A. Watson, Gabrielle B. Nowara, Klaas Hartmann, Bridget S. Green, Sean R. Tracey, Chris G. Carter

**Affiliations:** 1 Institute for Marine and Antarctic Studies, University of Tasmania, Taroona, Tasmania 7001, Australia; 2 EcoMarine MetaResearch, Sandy Bay, Tasmania 7006, Australia

## Abstract

The growing human population must be fed, but historic land-based systems struggle to meet expanding demand. Marine production supports some of the world's poorest people but increasingly provides for the needs of the affluent, either directly by fishing or via fodder-based feeds for marine and terrestrial farming. Here we show the expanding footprint of humans to utilize global ocean productivity to feed themselves. Our results illustrate how incrementally each year, marine foods are sourced farther from where they are consumed and moreover, require an increasing proportion of the ocean's primary productivity that underpins all marine life. Though mariculture supports increased consumption of seafood, it continues to require feeds based on fully exploited wild stocks. Here we examine the ocean's ability to meet our future demands to 2100 and find that even with mariculture supplementing near-static wild catches our growing needs are unlikely to be met without significant changes.

## Abstract (web summary)

Global landings of wild-caught seafood have plateaued in recent years. Analysing trends in global fisheries catches, Watson *et al.* find that distance between sourcing and consumption has increased steadily since the 1950s, with ocean productivity unlikely to meet current consumption rates by 2100.

## Graphical abstract

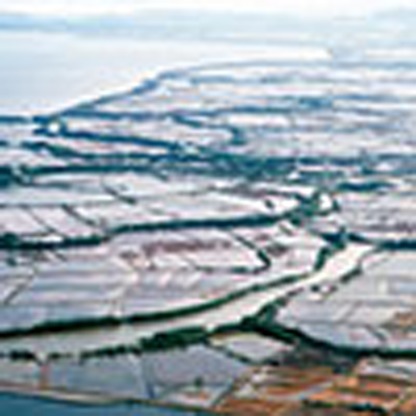

Through the history of humankind there has been a population-driven and technology-supported expansion in the use of the world's ocean as a source of protein^1^. By the time of the first reliable records some areas of the world's oceans had been fished for centuries and their bounty widely traded. Fishing expanded and intensified with technological development and a need to find new resources. Recent years, however, have seen recognition that global landings of wild-caught seafood have plateaued^2^. Many stocks have been fished to their maximum limits and unfortunately many beyond these limits, while other valuable sources of fish protein are in recovery mode^3^. The cost of over-harvesting has been considerable^4^,^5^.

Marine systems vary in their productivity but ultimately, without significant modification such as those terrestrial farming introduced, they are limited in what they can supply, especially with higher trophic species^6^ such as farmed salmon. By far, small pelagic fish constitute the largest biomass removed by current industrial fishing. Humans, however, only directly consume two-thirds of this, with the remainder used as fodder. Fodder fish, processed to meal and oils, have become valuable to our production of terrestrial livestock but have also facilitated the development of the mariculture production of carnivorous species such as prawns and salmons that are a popular and valuable seafood.

Future increase to seafood supply is now expected to come from the continued expansion of global mariculture. For this to happen fishmeals and oils rendered from wild populations of fodder species cannot be used as the basis of increased production. As it stands, two-thirds of current mariculture production requires formulated feeds with fishmeal additives and this has substantially increased from 1980 when it was only 50% (ref. 7). There is also increasing demand for fodder fish to be consumed directly by humans. To contribute to global food security any substitutes for fishmeal in feeds should not rely on sources that could be directly utilized by humans. Considerable advances in feed formulation predict a reduction in fishmeal inclusion in fish feeds is achievable^8^.

Future food security of the majority may depend on our oceans^9^. We show that the human footprint on global ocean production is large and growing. Many countries require areas multiple times the size of their own waters to support their population's seafood requirements. Fleets travel widely^10^ and seafood is traded globally in unprecedented volumes^11^. Our results demonstrate how incrementally each year, marine foods are sourced farther from where they are consumed. The proportion of the oceans' supporting marine primary productivity diverted to feed humans is large and increasing. Natural ocean systems have limitations and without major change we demonstrate that it is unlikely that our future requirements will be met.

## Results and Discussion

### Expansion of sourcing of marine foods

One measure of the expansion of our use of the world's oceans as our larder is the distance fishing fleets travel to meet our demands year upon year. Any such measure is strictly a minimum average value as seafood is a highly global commodity^7^, which can be caught in one location, processed at another and consumed in yet more distant places. Regardless of where it is caught, there is anecdotal evidence of seafood being processed where labour is the cheapest or where it can be rebadged to overcome import tariffs^12^.

**Figure 1:**
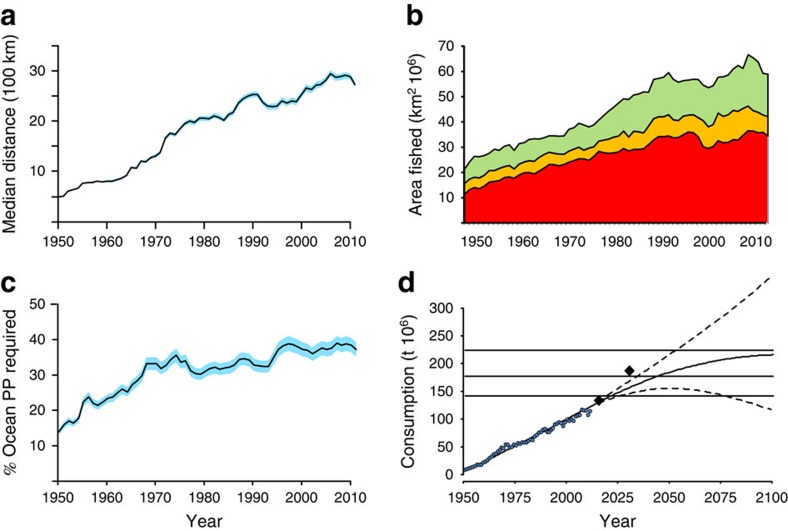
Historical and projected use of global oceans. (**a**) The median minimum distance 1950–2011 that seafood is sourced from where it is consumed (bootstrap methods supply 95% confidence limits indicated by blue shading) combines wild capture and mariculture using the mapped position of origin directly to the nearest port where consumed. (**b**) The area of ocean used to supply seafood for 1950–2011 using the % of annual primary productivity required (PPR) of the available primary productivity (PP) for three exploitation levels (>10% (green), >20% (yellow) and >30% (red)) assuming fixed trophic transfer efficiencies for the associated mapped landings. (**c**) Percentage of ocean PPR to PP used from currently accessible ocean areas (depth <1,000 m) assuming fixed trophic transfer efficiencies for the associated mapped landings for 1950–2011. Monte Carlo methods provided the 95% confidence shading in blue. (**d**) Global consumption of seafood 1950–2011 and projected to 2100 based on the UN's high, low and median levels of population estimates. Solid diamonds are FAO/UN's future consumption estimates. Horizontal lines represent the estimated limits to global seafood production (wild and farmed combined) assuming limits to the fishmeal (marine-sourced) input to mariculture feeds restricted to 10% (lowest line), 7 and 5%, respectively (highest line).

Any measure of distance that seafood travels to consumers must also accommodate the rapid rise of mariculture in the proportion of seafood consumed. Here we show (Fig. 1a) that the minimum mean distance travelled to source the seafood we consume has increased continually from the 1950s (when global catch records started^13^) to the present. At least some of this migration of fishing activities was also caused by increased controls of fisheries in declared exclusive economic zones (EEZs), first as a result of the United Nations Convention on the Law of the Sea in the 1970s, and subsequently through consistent establishment of management systems (with diverse success) all over the national fishing areas. Nevertheless the need to source seafood from increasingly longer distances is clear.

**Figure 2:**
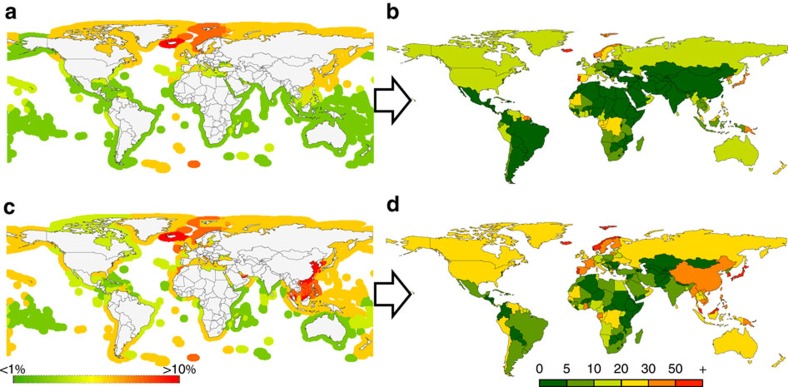
Where global seafood was historically produced and consumed. (**a**) Production in 1950s: the % of primary productivity required (PPR) of average primary productivity (PP) available to support seafood catches within country's exclusive economic zone (EEZ). (**b**) National consumption estimated for circa 1961 in kg per capita. (**c**) Production in 2000s: the % of primary productivity required (PPR) of average primary productivity (PP) available to support seafood catches within country's EEZ claims. (**d**) National consumption for 2009 in kg per capita.

Not all seafood taken is consumed locally. Most seafood now, whether wild or farmed, as we have shown, is sourced at great distances from where it is consumed. Country consumption levels for the early 1960s are shown in Fig. 2b. These are the consumers of the significant ocean production removed by fishing in areas mapped in Fig. 2a. Consumption was generally highest in countries that have long traditions of fishing and fish in the diet such as Norway, Iceland and Portugal. Most of these countries have large fleets that fish in the waters of other countries. Some countries do not have large fishing fleets but use imported seafood as a major protein source. By the 2000s (Fig. 2d) seafood consumption levels had generally risen and had greatly increased in Asia and Europe, which required their fleets to travel widely to maintain annual landings. Driven by demands for seafood, European fleets were fishing in the waters of countries of northwest Africa^10^ (see Fig. 2c), and China had deployed considerable distant-water fleets fishing throughout the Pacific and along the African coast^14^. The intensity and global nature of the seafood trade had greatly increased over this 50-year period^15^.

Some countries, including many developing ones, rely on production from freshwater. Food and Agriculture Organization (FAO) reported that from 2001 to 2011 there were 352M t reported for marine areas compared with 6M t for freshwater^7^. Since 1950 to 2011 capture landings from marine sources have risen fivefold but for freshwater it was 45-fold. They reported 30M t (32% of global total) produced from marine habitats (which we will refer to as mariculture) in 2010 as against 62M t for freshwater (62%) with the balance in brackish waters. Freshwater production, however, can be dependent on marine-based feeds.

### Increased use of ocean primary productivity

At the same time as we have sourced seafood from greater distances, we have utilized, through our harvested seafood and the mariculture it supports, an increasing proportion of calculated total annual ocean production. There is uncertainty associated with the calculation of ocean primary production using satellite ocean colour data such as that from SeaWIFS data used here, particularly in the most productive inshore and shelf areas where most seafood is taken. The area of ocean fished has increased (Fig. 1b), however, including those areas fished to an intensity that requires 30% or more of calculated *in situ* annual primary productivity to support it. A consideration of ocean areas that are accessible by most methods of fishing (those less than 1,000 m in depth) shows that we could be now using an average of nearly 40% of calculated ocean primary production in those areas (Fig. 1c). If, however, SeaWIFS data are underestimating primary production then this will be the worst-case scenario.

Most of the productive areas of the world's seas are near the coast, and it is here that 80% of the wild-caught seafood is taken. The intensity of use within the marine EEZs claimed by maritime countries for fishing varies, but globally this has increased greatly. In the 1950s (Fig. 2a) most tropical and southern hemisphere waters yielded annual landings that were supported by only a small proportion of local marine primary productivity. By the 2000s, fishing was taking a much larger proportion of available production (Fig. 2c) and had greatly intensified throughout Asia and South America. In all but a few countries, fishing extracted 5% or more of ocean production from their waters.

Taken together with the logistics of fishing, these natural limits to ocean productivity^6^ restrict the harvest of wild seafoods. Despite an increasing demand for valuable seafood the expansionary trends in the sourcing of wild seafoods shown are markedly slowing.

### Ability to meet future seafood demands

Global consumption of seafood has increased since the 1950s and, notwithstanding nature's limitations, is projected to continue (Fig. 1d). Here we show projections to 2100 from historical data based on UN population projections, which vary by assumed human fecundity^16^. Expert UN future estimates expect considerable increase in consumption rates^17^ (diamond-symbols Fig. 1d). Given the trend from the last decade of wild-caught seafood landings it is expected that catches will remain largely static^2^; therefore, expected increases in consumption are widely anticipated to come through significant expansion in mariculture. Feeding most farmed fish, including farming freshwater fish which is 60% of all fish production^7^, currently requires wild-caught marine landings. In addition, feeds produced from marine fodder fish are used for terrestrial livestock production^18^.

We estimate the limits to global human consumption possible if wild-caught seafood landings remains relatively static but mariculture is expanded as anticipated. Carnivorous fish species cannot use carbohydrates as an energy source, and their required feeds are very rich in proteins and oils, traditionally provided by fishmeal and fish oils, rich in essential fats. As nutritional knowledge increases for mariculture species the inclusion of fishmeal and oil can be reduced; this is reflected by our scenarios using 5, 7 and 10% fishmeal inclusion. The limit to future global seafood consumption of 144 million tonnes (lower horizontal line Fig. 1d) is our estimate if overall feeds used contain only 10% fishmeal originating from wild sources. The bulk of the feed would initially be sourced from terrestrial agriculture including a wide variety of plant and rendered animal sources^19^,^20^. If, however, the content of wild-sourced fishmeal in mariculture feeds is reduced to only 7% overall, then a large increase to 177M t annually is achievable (middle horizontal line Fig. 1d). With the dependence on wild fish stocks further reduced, whether transforming further agricultural production or through new technologies, these limits could be lifted allowing the projected increase in global consumption to continue. Global production (wild and mariculture combined) to 220M t could be achieved if the overall fishmeal content was dropped to 5% (top horizontal line Fig. 1d). This will, however, require significant change because, in fact, the trend has been to have an even greater reliance on natural ecosystems for feeds and not the reverse. A full two-thirds of mariculture production requires formulated feeds now which is a substantial increase from 1980 when it was only 50% (ref. 7). In the meanwhile global seafood requirements can only be met if the viability of wild stocks and their supporting ecosystem are not compromised. The health of our marine ecosystems and the stocks they support remains vital.

We anticipate also that climate change will alter both production and consumption patterns of seafood by changing the productivity of marine areas^1^,^21^,^22^, the use of coastal land and in other ways as yet unexpected. In one projection, anticipated climate change will increase wild productivity by 2050 by providing a 6% increase in larger species directly consumed as seafood, and nearly 4% more of smaller species currently used as fodder^1^. If somehow through incentives, we could also consume the estimated 7M t annually currently discarded at sea^23^, then a total of 200M t of seafood would be available. If this increased production was realized then the extra fodder fish captured, but not used for mariculture, could be used for human consumption or provide increased inputs to livestock production on land^18^. At 200M t, the anticipated consumption corresponding to the mean projection of global populations would be met until about 2050^1^, but after this time increases must come by further decoupling mariculture production from the limitations of natural marine ecosystems. Future feeds must not come from sources currently used to feed humans but from additional, currently untapped sources such as microbial or planktonic production. How then can we meet our future demands for seafood? Mariculture, and more broadly aquaculture, has potential to increase production through a variety of mechanisms; fish are effective at converting feeds to protein^8^ and improvements in nutrition will further improve this especially when combined with domestication and selective breeding. The availability of different protein and oil sources is increasing as key ingredients are refined, into protein concentrates for example, and new ones introduced, such as from insects and algae. Many mariculture species have been farmed for only a few generations and there is still much potential for selective breeding. For example, some individual carnivorous trout are better at using plant proteins while other individuals may be better at retaining valuable omega-3 fatty acids^20^. Atypical marine species, such as Senegalese sole that synthesize long-chain omega-3 fatty acids, may have valuable characteristics and be preferred.

Societal choice will influence future directions; genetically modified (GM) salmon that grow much faster were developed over 15 years ago and GM plants that produce high levels of long-chain omega-3 oils will be available in the next few years^19^. Our future may see salmon mariculture come onshore in recirculation systems and even stay in freshwater to reduce stress and disease while increasing growth efficiency. Integrated multi-trophic level marine systems that recycle wastes from farmed fish through harvestable crops such as seaweed, grazing abalone and filter feeding bivalves will expand.

In addition to our vital wild captures and harvest, seafood of the future is likely to be produced in a variety of ways. For basic food requirements there will be more production of marine plants (GM or otherwise). These could be in shallow coastal areas but they could be inland or even in suspension in huge volumes of seawater. In addition, proteins will be produced by growing a range of animals on massive scales without regard to omega-3 oil content or similar current limitations. Required feeds could be comprised of plants, invertebrates or even microorganisms. Production will include a variety of filter feeders and benthic detritivores. Although initial consumer acceptance is uncertain, we expect that GM plants will allow the production of seafood with high omega-3 oil content. Finally, there will be the niche market that most consumers associate with current seafood production, which will be limited and expensive. This will consist of growing selectively bred species like salmon on natural oil-rich feeds. We expect the seafood of the future, wild and farmed, to be even more diverse than what is available today.

Climate change will rearrange ocean productivity^21^, and increasing populations and limitations on terrestrial agriculture^9^,^24^,^25^ will increase our demands on the world's oceans, with the burden of change largely passed to the poor^26^. Coastal areas may be abandoned with increased flooding; however, their use for marine and brackish water food production will likely be increased.

The maximum potential of the world's oceans to feed us is the focus of much current research activity. There are a range of solutions proposed that could be pursued. These include recovering overfished stocks and their productivity^3^,^27^, making better use of what we do harvest and reducing waste starting from discarding at sea to spoilage through distribution chains^2^,^23^. The scale of response needed requires much more support of the United Nations and other bodies that transcend national limitations as they strive to ensure better adaptation of international instruments such as the Port State Measures Agreement^7^.

As populations demand more marine-sourced food production and while seafoods remain highly sought-after by wealthy nations choices must and will be made. Markets and society in general will decide which production systems consume finite resources such as water, energy, coastal areas and our essential but ultimately limited wild ocean inputs.

## Methods

### Catch and seafood data sources

Mapped fisheries landing data 1950–2011 was prepared from the FAO data supplemented by regional data sets^13^. Global seafood import and export data were sourced from FAO^11^.

### Minimum distance for seafood sourcing

Global seafood export records were matched to mapped catches using hierarchal fuzzy-fit methods so that the location of capture of exported seafood could be identified. These results, which formed a virtual seafood global market place, were then matched with global import records. This allowed the provenance of wild-caught seafood to be determined. The direct (great-circle) distance between the nearest port in the importing country (or adjacent to it) and the 30-min spatial cell where the seafood was caught was calculated. The global median minimum distance used catch-weighted (tonnage) records for each year and country. Seafood produced by mariculture from FAO's records was also included in the distance averaging. For imported mariculture we used the minimum distance between any coastal 30-min spatial cell in the country of origin and any port in the importing country. Domestically produced mariculture was also included but a zero distance was assigned for this fraction. To determine some likely confidence limits to the median sourcing distance trend we bootstrapped samples of records in a hierarchal manner. For each of 1,000 trials for years 1950 to 2011 (inclusive), the bootstrapping process sampled 10,000 seafood distance records. It was first decided, based on probabilities of published total tonnages, whether the seafood was wild-caught by the country consuming it, imported wild-caught seafood, imported mariculture seafood or domestic mariculture. Within a category an appropriate national minimum distance was used in a catch-weighted estimate. We examined the impact of the variation between modes of sourcing and between-country variability by ranking the results and selecting the values corresponding to the 5% confidence limits. Bootstrap sampling of these sources and national averages was used to produce 95% confidence limits. Not all annual estimates were normally distributed when tested (Shapiro-Wilk)^28^; therefore, the median values were plotted.

### Area of the ocean providing seafood

Mapped wild-caught seafood (which also provides significant fodder for fishmeal used in mariculture)^20^ was used to determine how much area of the global oceans is utilized. To estimate the intensity of utilization we calculated the ratio between the primary productivity required to produce the annual seafood landings (at the appropriate trophic level) and the average primary productivity from a 10-year average of satellite data (SeaWiFS, http://seawifs.gsfc.nasa.gov/ accessed 31 October 2012). Areas based on 30-min spatial cells in which a minimum of 10% of local primary productivity is required (PPR) annually to produce extracted seafood are shown in km^2^ × 10^6^, split into three bands. The top band (green) shows the area using >10% but <20%. The middle band (yellow) shows the area where PPR was >20% but <30%. The darkest bottom band (red) shows the area where PPR was >=30%.

### Source and sink of global seafood by countries and their waters

Most coastal countries claim an EEZ where they control fishing access. For each country we determine the percentage of average (1998–2007) primary productivity (based on SeaWIFS data) in their EEZ claim of the primary productivity required (PPR) to produce the seafood taken from their waters based on the trophic level of the seafood. This allows the source of seafood production globally to be mapped for the 1950s and the 2000s. We mapped for circa 1961 and 2009 the per capita consumption (kg) of seafood. This allows the relative seafood consumption demands of countries to be examined and put into context with each country's capacity to produce wild seafood. This establishes the source and sink of global seafood and how it has changed with time.

### Percentage of accessible ocean's primary productivity used to supply seafood

The PPR^29^ overall and by EEZ area was computed from


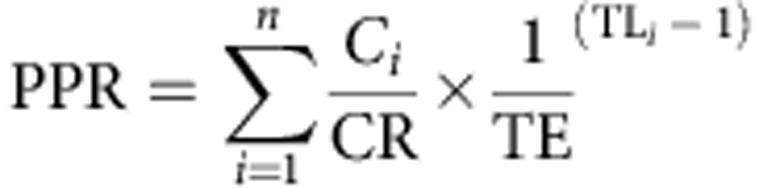


where *C*_*i*_ is the catch of species *i*, CR is the conversion rate of wet weight to carbon, TE is the transfer efficiency between trophic levels, TL_*i*_ is the trophic level of species *i* and *n* is the number of species caught in a given area. We applied a 9:1 ratio for CR and a 10% value was used for TE^29^ (Monte Carlo (MC) analysis assumed 90% CL from 5 to 15%, and varied this from 1.5 to 20%). Species-specific trophic levels were taken from FishBase ( www.fishbase.org) for fishes and SeaLifeBase ( www.sealifebase.org) for invertebrates. Primary production estimates 1998–2007 based on satellite data (SeaWiFS, http://seawifs.gsfc.nasa.gov/ accessed 31 October 2012). MC methods explored 95% limits by varying annual PP estimate and TE (from 5 to 15%). Spatial resolution is limited for upper trophic levels and highly migratory species.

The percentage of accessible global ocean primary productivity used through fishing annually was estimated as above. This used only accessible fishing areas where depths are <1,000 m (typically within coastal maritime claims). Monte Carlo methods (in *R*) were used to estimate the sensitivity of estimates to assumptions about (1) global primary productivity and (2) the transfer efficiency between tropic levels. For each year 1,000 trials were completed by randomly selecting a value for global primary productivity from a normal distribution based on the mean and s.d. of 10 available annual SeaWIFS data sets 2007–2011 (SeaWiFS, http://seawifs.gsfc.nasa.gov/ accessed 31 October 2012) selected at random to represent available primary productivity. For each trial a transfer efficiency between tropic levels was chosen from a normal distribution with a mean of 10% and a standard distribution of 3.03 representing the approximate 90% confidence limits for a range from 5 to 15%.

### Global consumption of past and projected seafood

A GLM (in R) model of global seafood consumption was fitted to historical population^16^ ( http://www.ggdc.net/maddison/oriindex.htm accessed 5 February 2014) estimates and extended using a linear projection of UN fecundity scenarios^16^. Seafood supply limits were calculated based on parameters from FAO^30^. Seafood consumption levels were also sourced from FAO.

Future estimates of consumption based on FAO are shown as diamonds. Supply limits to seafood were calculated based on parameters in Table 1, Seafood is defined as food whose production is dependent on marine ecosystems. Seafood can be wild caught or farmed through mariculture.


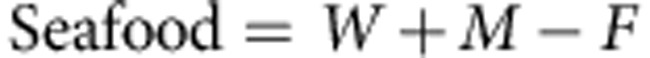


where *W* is 105 Mt calculated as the sum of average (2005–2011)^13^ of reported global wild catch landings of 79M t and an estimate of 26M t of illegal and unreported landings^31^. This total is assumed nearly stagnant (see Table 1). We allowed wild capture (both reported and unreported landings) to drop 10% but to increase 20% from recent levels^13^,^31^. Current levels of wild capture landings used for fishmeal processing were assumed^32^. We assume an optimistic conversion efficiency of 100% for farmed fish to allow for farming of filter feeders^33^. Seafood consumption levels were sourced from FAO^7^.

All weight captured or produced is used for direct human consumption or as fodder, *M* is the mariculture production and *F* is the tonnage removed for fodder calculated as


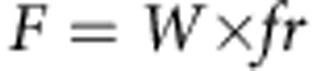


where *fr* is proportion of wild catch used for fodder and not for seafood. Mariculture production *M* is dependent on *F* as


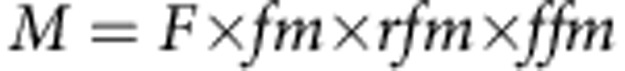


where *fm* is the proportion of fodder used in fishmeal, *rfm* is the reduction rate of fodder fish to fishmeal (and edible oils), *ffm* is the proportion that fishmeal makes up of the feeds used in mariculture (when blended with other ingredients) (varies by species and we examined 5, 7 and 10% overall). We assume an optimistic conversion efficiency of 100% (ratio of fish produced to feed used) to allow for filter feeder production.

A sensitivity analysis was conducted on the seafood production using the ranges in Table 1 for each of the three scenarios of proportion of mariculture feeds that is fishmeal (*ffm*). From 1,000 simulations the total range of 95% confidence limits production was never >2% of the mean in any *ffm* scenario. The output was normally distributed and therefore is represented by the mean.

We show the limit to seafood production (wild and mariculture combined) based on an average of 5, 7 and 10% fishmeal in feeds (*ffm*). Population scenarios used for the projection of seafood consumption needs came from the United Nations^16^.

## Additional information

**How to cite this article:** Watson, R. A. *et al.* Marine foods sourced from farther as their use of global ocean primary production increases. *Nat. Commun.* 6:7365 doi: 10.1038/ncomms8365 (2015).

## Figures and Tables

**Table 1:** Values of parameters used to calculate possible wild and mariculture seafood production.

**Name**	**Mean**	**Min**	**Max**	**Description**
*W*	105	94.5	126	Wild capture (reported and IUU landings)—range allows a 10% drop and up to 20% increase^13^,^31^
*fr*	0.36	0.25	0.45	Proportion of wild used for non seafood^32^
*fm*	0.9	0.7	1.0	Proportion of fodder used in fishmeal^32^
*rfm*	0.22	0.21	0.24	Reduction rate fodder to fishmeal^18^
*ffm*		0.05	0.1	Proportion of mariculture feeds that is fishmeal/oils as per scenarios (10, 7 and 5%)
